# Gene Duplication, Shifting Selection, and Dosage Balance of Silicon Transporter Proteins in Marine and Freshwater Diatoms

**DOI:** 10.1093/gbe/evad212

**Published:** 2023-11-23

**Authors:** Karolina Bryłka, Eveline Pinseel, Wade R Roberts, Elizabeth C Ruck, Daniel J Conley, Andrew J Alverson

**Affiliations:** Department of Geology, Lund University, Lund, Sweden; Department of Biological Sciences, University of Arkansas, Fayetteville, AR, USA; Department of Biological Sciences, University of Arkansas, Fayetteville, AR, USA; Department of Biological Sciences, University of Arkansas, Fayetteville, AR, USA; Department of Geology, Lund University, Lund, Sweden; Department of Biological Sciences, University of Arkansas, Fayetteville, AR, USA

**Keywords:** codon models, episodic selection, gene duplication, ohnolog, relaxed selection

## Abstract

Numerous factors shape the evolution of protein-coding genes, including shifts in the strength or type of selection following gene duplications or changes in the environment. Diatoms and other silicifying organisms use a family of silicon transporters (SITs) to import dissolved silicon from the environment. Freshwaters contain higher silicon levels than oceans, and marine diatoms have more efficient uptake kinetics and less silicon in their cell walls, making them better competitors for a scarce resource. We compiled SITs from 37 diatom genomes to characterize shifts in selection following gene duplications and marine–freshwater transitions. A deep gene duplication, which coincided with a whole-genome duplication, gave rise to two gene lineages. One of them (SIT1–2) is present in multiple copies in most species and is known to actively import silicon. These SITs have evolved under strong purifying selection that was relaxed in freshwater taxa. Episodic diversifying selection was detected but not associated with gene duplications or habitat shifts. In contrast, genes in the second SIT lineage (SIT3) were present in just half the species, the result of multiple losses. Despite conservation of SIT3 in some lineages for the past 90–100 million years, repeated losses, relaxed selection, and low expression highlighted the dispensability of SIT3, consistent with a model of deterioration and eventual loss due to relaxed selection on SIT3 expression. The extensive but relatively balanced history of duplications and losses, together with paralog-specific expression patterns, suggest diatoms continuously balance gene dosage and expression dynamics to optimize silicon transport across major environmental gradients.

**Significance**

Many factors shape the evolution of protein-coding genes and gene families. For example, their function can be optimized by adaptive mutations that change an amino acid or by altering gene expression dynamics through gene duplications or losses. These questions were explored in diatoms, a group of microeukaryotes encased by silicon (glass) cell walls. The proteins that mediate the import of dissolved silicon, called SITs, have an extensive and ongoing history of gene duplication and loss. Numerous sources of data together suggest that optimization of gene expression has played a central role in shaping sequence evolution and gene family dynamics of diatom SITs.

## Introduction

Natural selection on protein-coding genes can change in both strength and type in the wake of environmental changes, such as movement into new habitat, or genomic changes, such as a gene or genome duplication. By providing new mutational substrates for natural selection to act upon, gene duplications have long been recognized as an important source of evolutionary innovation. Although most duplicates confer no functional benefits and are lost through pseudogenization (Lynch and Conery 2000), surviving duplicates can retain their ancestral function (redundancy), evolve a modified function (subfunctionalization), or, in some cases, evolve new functions (neofunctionalization) (Ohno 1970). Each outcome involves a distinct mode of natural selection, measurable by codon models that can detect relaxation of selection, maintenance through purifying selection, or functional divergence through diversifying selection (Yang and Nielsen 2002). In the absence of evidence for adaptive evolution at the sequence level, some genes with strong environmental associations show increased rates of duplication and loss (Clark et al. 2007). The probability that a duplicated gene is retained depends, in part, on dosage effects and whether the duplicate originated from a small-scale or whole-genome duplication (Lynch and Conery 2000; Maere et al. 2005). Similar shifts in the selective regimes will operate as organisms diversify in new habitats as well. Surface proteins in microbes, in which the entire organism interfaces directly with its surroundings, should be especially sensitive to changes in the environment.

Diatoms are one of Earth's foremost primary producers and one of a few lineages with cell walls composed of rigid silica (Round et al. 1990). Diatoms represent one of the largest biological sinks of environmental silica (Tréguer and De La Rocha 2013), an element that comprises roughly one-quarter of Earth's crust (Iler 1979). Diatoms take up dissolved silicon (DSi) in the form of orthosilicic acid, which is scarce (≍10 μM Si) across most of the ocean (Frings et al. 2016). DSi concentrations are generally much higher (85–100 μM Si) in freshwater lakes and rivers (Frings et al. 2014, 2016). At high DSi concentrations, typical of many freshwater environments, diatoms can import DSi passively through diffusion, whereas at low and potentially growth-limiting concentrations, typical of marine environments, DSi is actively imported by silicon transporter (SIT) proteins (Thamatrakoln and Hildebrand 2008; Hildebrand et al. 1997). Environmental DSi concentrations appear to impact how diatoms acquire and use silicon. Compared with freshwater diatoms, marine diatoms use less silica (Conley et al. 1989) and have a greater enzymatic affinity for DSi (Martin–Jézéquel et al. 2000), which might be expected in environments where it is scarce (Alverson 2007).

SITs are a gene family composed typically of 3–5 gene copies in most species, with phylogenetic studies highlighting a dynamic history of recurrent and often recent gene duplications and losses (Alverson 2007; Thamatrakoln et al. 2006; Durkin et al. 2016). Experimental evidence has revealed paralog-specific expression associated with cell cycle progression (Hildebrand et al. 1998; Sapriel et al. 2009; Thamatrakoln and Hildebrand 2007), suggestive of subfunctionalization following gene duplication (Baker et al. 2013). In the diatom *Cyclotella nana*, transcript levels of two SITs (SITs 1 and 2) were highest during the S phase of the cell cycle, whereas transcription of a third copy (SIT3) was uniformly low throughout the cell cycle (Thamatrakoln and Hildebrand 2007). Paralog-specific patterns of SIT expression have been found in other diatoms as well (Hildebrand et al. 1998; Sapriel et al. 2009). Taken together, evidence across several diverse diatoms suggests that gene duplication and functional differentiation, particularly in gene expression, have played important roles in the evolution of diatom SITs, with important consequences for silicon metabolism (Durkin et al. 2016; Martin–Jézéquel et al. 2000). There is less evidence for adaptive evolution at the sequence level, where diatom SITs have been shown to evolve predominantly under strong purifying selection (Thamatrakoln et al. 2006; Alverson 2007).

One diatom lineage, Thalassiosirales, is common and diverse throughout marine and freshwater systems (Alverson et al. 2007; Roberts et al. 2023). The availability of dozens of sequenced genomes allowed us to comprehensively sample SITs across the lineage and test specific hypotheses about changes in selection following gene duplications and marine–freshwater transitions. The results highlight the power of a comprehensive, genome-derived data set to uncover the evolutionary dynamics of an ecologically and metabolically important gene family.

## Results

A total of 117 SIT sequences were assembled from the draft genome sequences of 37 Thalassiosirales (25 marine and 12 freshwater) and several outgroup transcriptomes (Roberts et al. 2023). After rooting the gene tree and removing the most distant outgroups, the alignment included SITs from 108 Thalassiosirales and one outgroup species, *Eunotogramma lunatum*.

### SIT Topology Prediction

**Fig. 1.**
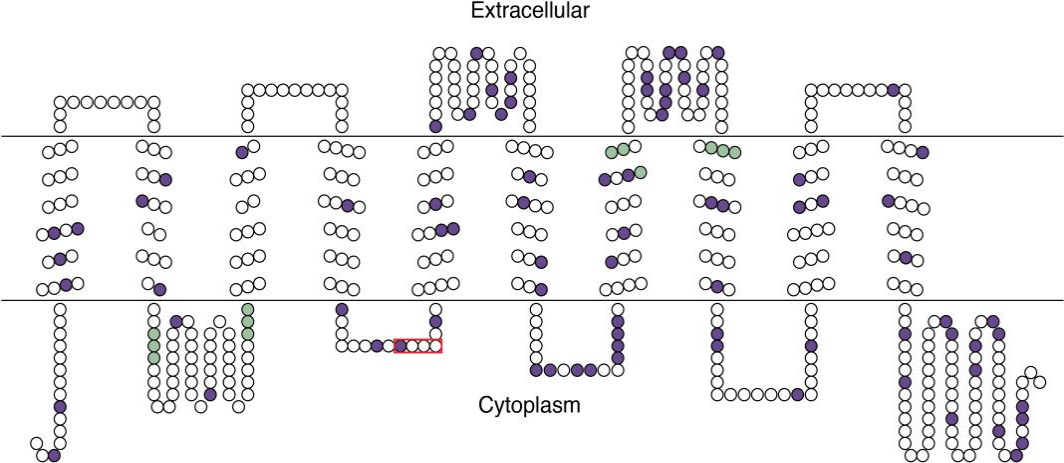
Reference SIT topology prediction from *Skeletonema grethae*. Amino acid residues are shown by circles, and sites under episodic selection based on HyPhy's MEME model (*P* ≤ 0.05) are in dark purple. GXQ motifs are in light green and the CMLD motif is outlined in a red box.

We predicted the structure of all SITs in our data set using Consensus Constrained TOPology (CCTOP) prediction (Dobson et al. 2015). We used the prediction with the best reliability score calculated by CCTOP as a reference (fig. 1). Consistent with a previous study (Durkin et al. 2016), CCTOP predicted ten transmembrane domains (TMDs) without coiled-coil motifs (fig. 1). Two functional motifs thought to function directly in DSi uptake were present. Four GXQ (where X = Q, G, R, M) motifs were located in their expected and conserved positions (Hildebrand 2008): two on the inside loop between TMDs 2 and 3, one in TMD 7, and one in TMD 8. A single CML(I)D motif was also located in the expected and conserved position (Sherbakova et al. 2005; Grachev et al. 2005): between TMDs 4 and 5.

### SIT Duplication and Loss

**Fig. 2.**
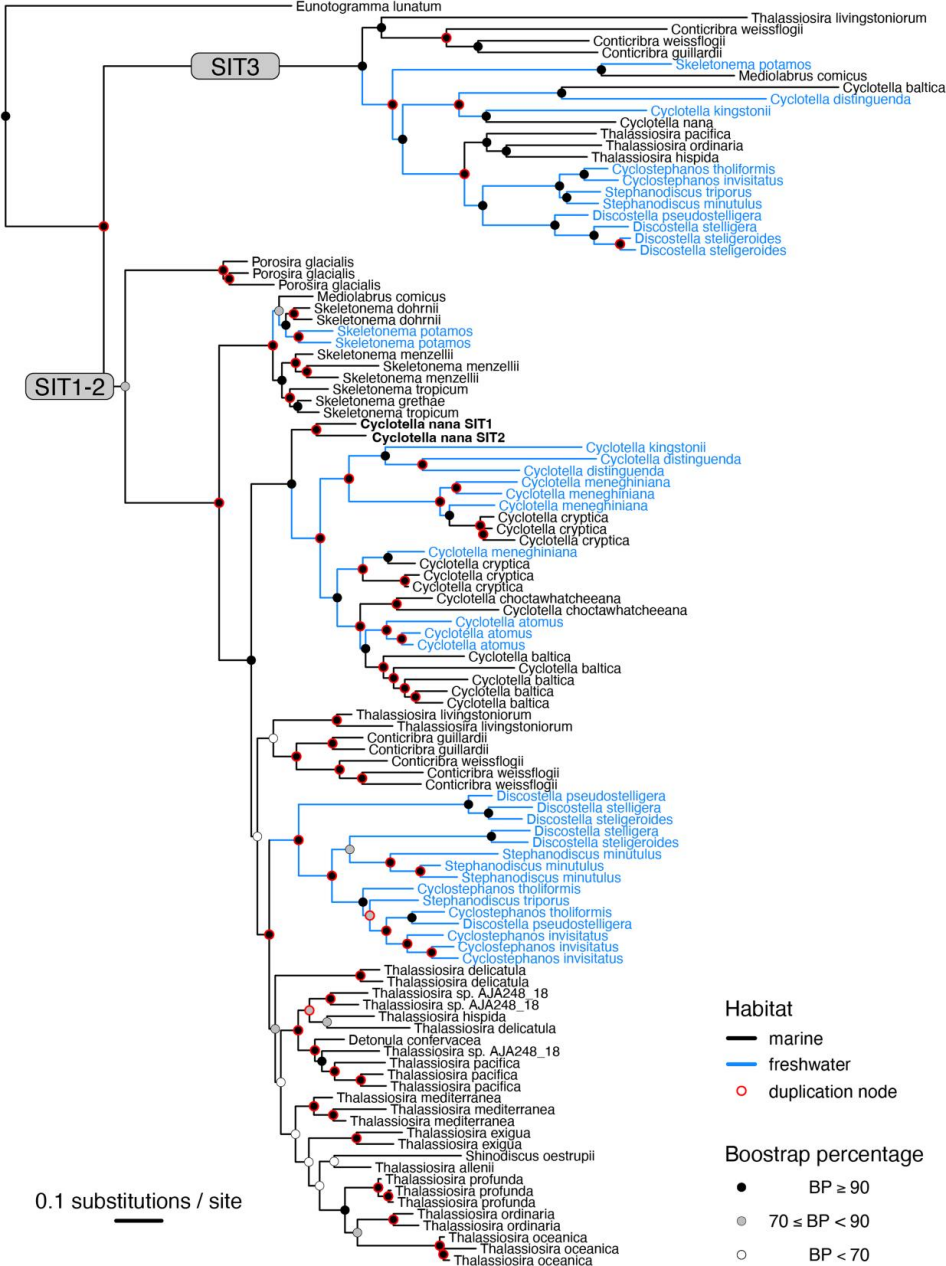
Phylogeny of SIT genes from marine (black) and freshwater (blue) Thalassiosirales. More distant outgroups were used to root the tree but were removed here. Duplications are marked by red outlines on the bootstrap labels. Bootstrap values are based on ultrafast bootstrap analysis implemented in IQ-TREE (Hoang et al. 2018).

Previous studies revealed an extensive history of gene duplication and loss in diatom SITs (Durkin et al. 2016; Thamatrakoln et al. 2006; Alverson 2007). The draft genomes of 37 Thalassiosirales contained 1–6 SITs, reflecting a dynamic history of gene duplication and loss. Reconciliation of the gene and species trees with NOTUNG estimated 62 gene duplications (fig. 2) and 72 losses over the course of Thalassiosirales evolution.

We identified two main clades in the gene tree, which we labeled based on SIT annotations in the model species, *C. nana*, where SIT expression and function have been well characterized (Thamatrakoln and Hildebrand 2007). One clade contains *C. nana* SITs 1 and 2 (“SIT1–2”) and the second contains SIT3 (“SIT3”) (fig. 2). All 37 Thalassiosirales species possessed at least one SIT1–2, but fewer than half the species (*n* = 18) possessed SIT3 (fig. 2). Most species had multiple SIT1–2 paralogs (range: 1–6 copies), but only two species had more than one SIT3 gene.

**Fig. 3.**
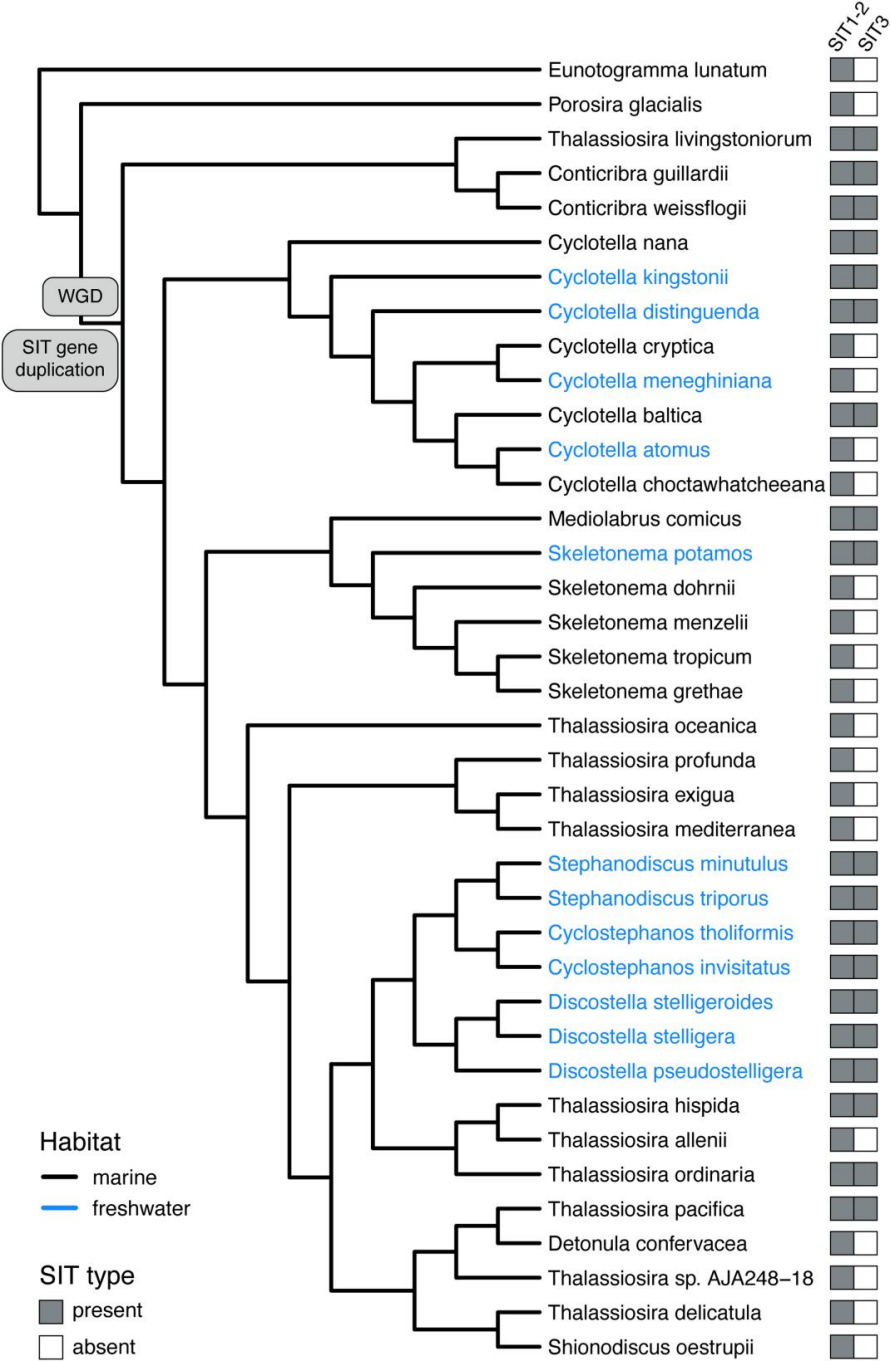
Species phylogeny of Thalassiosirales, modified from Roberts et al. (2023). Branch labels identify the putative time point of a gene duplication that resulted in the descendant SIT1–2 and SIT3 lineages (‘SIT gene duplication’) as well as a previously inferred whole-genome duplication event (‘WGD’) (Parks et al. 2018). Marine taxa are in black and freshwater taxa are blue.

A deep duplication led to the divergent SIT1–2 and SIT3 clades (fig. 2), which likely occurred after the split between *Porosira* and other Thalassiosirales (fig. 3) and coincided with a previously inferred whole-genome duplication event (Parks et al. 2018). Following this duplication, the history of SIT3 was dominated by losses, including 12 deep and 7 species-specific gene losses identified by NOTUNG (table 1). NOTUNG identified a total of five SIT3 duplications, one of which was species-specific (table 1, fig. 2). The SIT1–2 clade had a more balanced history of duplication and loss, with 56 duplications and 53 losses (table 1). Most of the SIT1–2 duplications and losses were species-specific, mapping to terminal branches on the species tree (table. 1, fig. 2). NOTUNG results are summarized in supplementary table S1 and figure S1, Supplementary Material online.

**Table 1:** The Number of Gene Duplications and Losses in the SIT1–2 versus SIT3 Clades (fig. 2), Based on NOTUNG Reconciliation of the SIT Gene Tree (fig. 2) and Thalassiosirales Species Tree (fig. 3)

	SIT1–2	SIT3
Total duplications	56	5
Internal node duplications	17	4
Leaf node duplications	39	1
Total losses	53	19
Internal node losses	24	12
Leaf node losses	29	7

### Molecular Evolution

We fit a range of codon models to test specific hypotheses of positive or relaxed selection in the context of gene duplications and marine–freshwater transitions, the results of which are summarized in table 2.

**Table 2:** Summary of Codon-Based Tests of Selection on Diatom SITs Using the HyPhy Software Program (Kosakovsky Pond et al. 2005)

Method	Selection Level	Test Branches	Background	*P* Value	#Sites Under Selection	Selection
BUSTED	Site	All	—	0	≥1	Positive
MEME	Site	All		≤0.01	45	Episodic
MEME	Site	All		≤0.05	81	Episodic
FEL	Site	All		≤0.01	464	Purifying

Method	Selection Level	Test Branches	Background	*P* Value	*K*	Selection
RELAX	Branch	SIT3 clade	SIT1–2 clade	0	0.62	Relaxed
		FW SIT1–2	M SIT1–2 (ex. *Eunot*. and SIT3)	0	0.57	Relaxed
		*Disc.* *+* *Steph.* *+* *Cyclost.* clade	M SIT1–2 clade	0	0.77	Relaxed
		*C. invisitatus* SIT3	SIT3	0.003	38.42	Intensified

### Tests of Positive Selection

The Fixed Effects Likelihood (FEL) method, which fits a site model with no variation in the strength of selection across the gene tree (Kosakovsky Pond and Frost 2005), revealed strong purifying selection in 93% of sites, with no sites under positive selection (*P* = 0.01). Against a predominant signal of purifying selection, site methods like FEL can fail to detect sites that have experienced instances of episodic diversifying selection on a subset of branches (Murrell et al. 2012). The Branch–Site Unrestricted Statistical Test for Episodic Diversification (BUSTED) test provides a general view of gene-wide episodic positive selection (Murrell et al. 2015) and suggested that one or more sites on at least one branch had experienced positive selection (*P* < 0.05). We followed up on this result with the more sophisticated Mixed Effects Model of Evolution (MEME) method to test for episodic selection (Murrell et al. 2015). Depending on the significance threshold, MEME identified 45 sites (9%) (*P* ≤ 0.01) to as many as 81 sites (16%) (*P* ≤ 0.05) that had experienced episodic diversifying selection. Positively selected sites were detected on internal and terminal branches, freshwater and marine branches, and on branches in both the SIT1–2 and SIT3 clades. Mapping positively selected sites onto the SIT protein structure (fig. 1) showed that positively selected residues were positioned throughout the protein, including transmembrane segments and inner and outer loops (fig. 1). A chi-square test showed that positively selected sites did not differ from expected proportions based on the total number of sites in transmembrane segments, inner loops, and outer loops in the SIT protein (fig. 1) (df = 2, *N* = 3, *P* = 0.72).

### Test of Relaxed Selection on SIT3

Given the uncertain role of SIT3 as a SIT (Thamatrakoln and Hildebrand 2007) and its widespread loss across Thalassiosirales (figs. 2 and 3), we tested whether purifying selection was relaxed in the SIT3 gene lineage (fig. 2). For this analysis, we set the SIT3 clade as the foreground branches and the SIT1–2 clade as the background (supplementary fig. S2, Supplementary Material online). The *Euntogoramma lunatum* outgroup sequence was excluded from all RELAX analyses due to its long branch length. RELAX indicated that selection was relaxed in the SIT3 clade compared with SIT1–2 (*P* = 0, *k* = 0.62). The threshold separating relaxation versus intensification of selection is *k* = 1, so a value of *k* = 0.62 is consistent with modest yet significant relaxed selection.

### Tests of Relaxed Selection in Freshwater SITs

Based on the vastly lower DSi concentrations in marine environments, we hypothesized that the strong predominant signal of purifying selection in the SIT1–2 clade was relaxed in freshwater diatoms, a hypothesis that was previously rejected but was not as directly testable with models available at the time (Alverson 2007). We focused here on the SIT1–2 clade because these SITs are known to actively import DSi (Thamatrakoln and Hildebrand 2007). For one test, the freshwater cyclostephanoid (*Discostella* + *Cyclostephanos* + *Stephanodiscus*) SIT1–2 clade was set as the foreground, and the rest of the SIT1–2 clade was set as the background (supplementary fig. S3, Supplementary Material online). The RELAX test supported relaxed selection in the cyclostephanoid SIT1–2 clade (*P* = 0, *k* = 0.77). For a second test, we expanded the set of reference branches to include all freshwater SIT1–2 branches in the foreground, with marine SIT1–2 branches as the background (supplementary fig. S4, Supplementary Material online). This test again found evidence for slight but significant relaxed selection in freshwater SITs (*P* = 0, *k* = 0.57).

### Divergent Transcriptional Profiles of SIT Paralogs

**Fig. 4.**
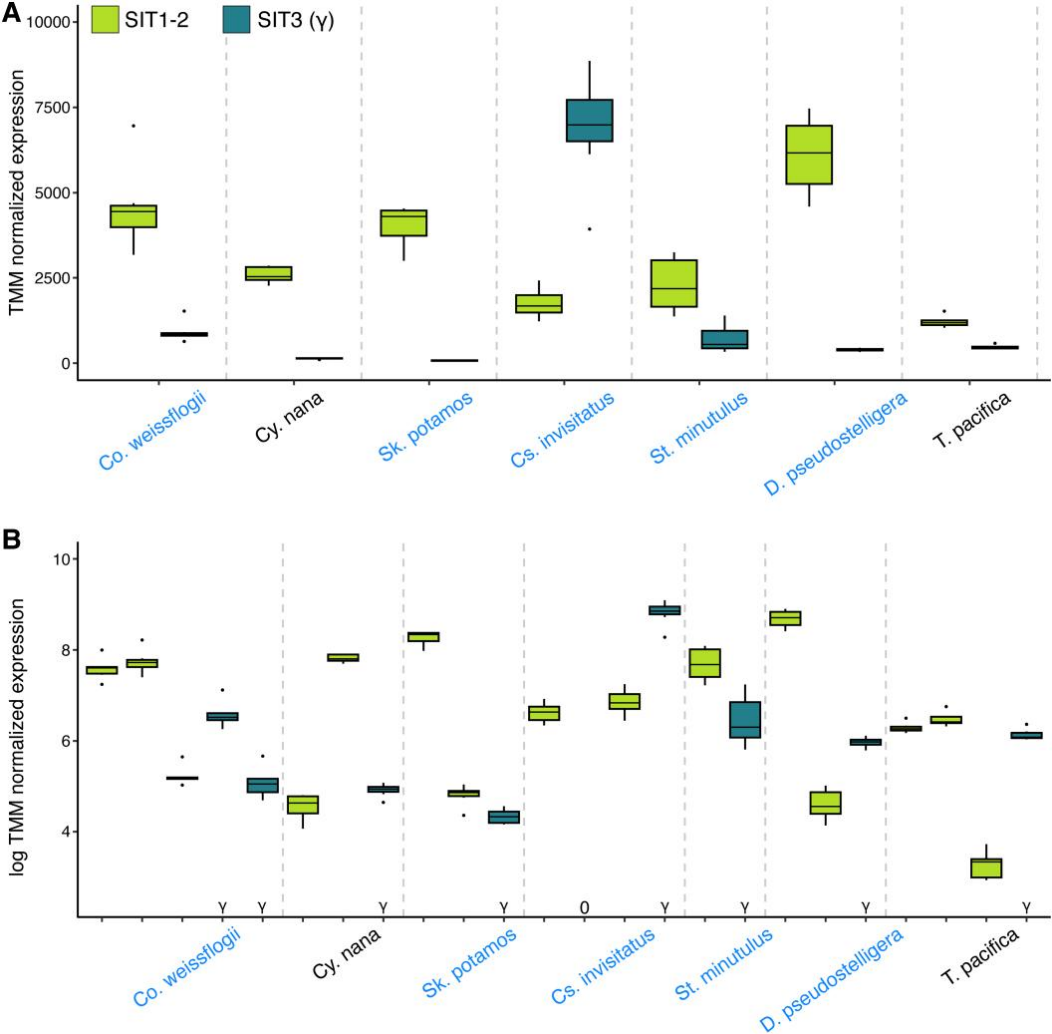
Transcriptional profiles of divergent SIT paralogs in diatoms, with transcript levels summed across paralogs for SIT1–2 versus SIT3 (*a*) or transcript level per paralog (*b*). In (*b*), unexpressed paralogs are labeled “0' and SIT3 paralogs are labeled “γ”. For clarity, transcript levels were log-transformed in panel (*b*) to see paralog-specific differences, which were more extreme than the summed differences shown in (*a*). Marine taxa are in black and freshwater taxa are in blue. Genus abbreviations: *Conticribra* (Co), *Cyclotella* (Cy), *Cyclostephanos* (Cs), *Discostella* (*D*), *Skeletonema* (Sk), *Stephanodiscus* (St), and *Thalassiosira* (*T*).

Previous work on the model species, *C. nana*, showed that transcript levels of SIT1 and SIT2 vary across the cell cycle, with peak expression during the S phase, whereas SIT3 is transcribed at uniformly low levels throughout the cell cycle (Thamatrakoln and Hildebrand 2007). We measured transcript levels for several species grown in batch culture under ideal conditions with replete silicon, and similar to the low-silicon conditions studied by Thamatrakoln and Hildebrand (2007), the summed transcript levels of SIT1–2 paralogs exceeded the SIT3 levels for all but one species (fig. 4*a*). Although the cultures in our experiment were not synchronized, our result on the model *C. nana* strain (CCMP1335) used by Thamatrakoln and Hildebrand (2007) recovered the same pattern of higher overall SIT1–2 levels relative to SIT3 (fig. 4*a*). Interestingly, one freshwater species, *Cyclostephanos invisitatus*, showed the opposite pattern, with consistently higher SIT3 than SIT1–2 transcript levels across all eight experimental replicates (fig. 4*a*). We followed up on this result by using the RELAX test to ask whether selection had been intensified in SIT3 of *C. invisitatus* (foreground) against the background of the rest of the SIT3 clade (supplementary fig. S5, Supplementary Material online), which we had previously found to be under relaxed selection compared with SIT1–2. This test showed that selection was greatly intensified on the *C. invisitatus* SIT3 branch (*P* = 0.003, *k* = 38.4), providing further evidence for greater functional significance of SIT3 in this one species. Finally, all species included in the experiments had two or more copies of SIT1–2, and in all cases, we detected substantial differences in transcript levels between one or more SIT1–2 paralogs, consistent with paralog-specific expression in these conditions (fig. 4*b*).

## Discussion

The SIT proteins of diatoms play an integral role in how they acquire and use silicon, the essential component of their cell walls and a growth-limiting nutrient in some parts of the ocean (Krause et al. 2019). Silicon levels are generally much higher in freshwaters, and several lines of evidence suggest that external silicon concentrations have cascading effects on many aspects of diatom physiology (Olsen and Paasche 1986; Conley et al. 1989; Martin–Jézéquel et al. 2000). The availability of dozens of sequenced genomes from a single lineage allowed us to ask new questions about SIT evolution in diatoms, including about the roles of gene duplication and loss and shifts in selection following gene duplications and marine–freshwater transitions.

### Two Distinct Lineages of SITs

By virtue of the genomic data used here, it is clear that a previous PCR-based SIT study failed to amplify and sequence the divergent SIT3 paralog (Alverson 2007) which, at that time, was known only from the sequenced genome of *C. nana* (Armbrust et al. 2004). As a result, the gene duplication history presented by Alverson (2007) was based exclusively on the SIT1–2 lineage, and although that history largely mirrored the SIT1–2 history reported here, it represented only one half of the SIT gene phylogeny.

The deepest SIT duplication occurred early on in Thalassiosirales and gave rise to two lineages that differ in sequence and possibly function. The timing of this duplication coincided with an inferred whole-genome duplication in Thalassiosirales (Parks et al. 2018), so although most SIT paralogs originated through small-scale duplications, the SIT3 lineage appears to have arisen through a different mechanism. The SIT1–2 and SIT3 gene lineages are putative ohnologs, duplicates that trace their origin to a genome duplication, giving them equal time to accrue mutations and evolve new functions (Wolfe 2000). Whole-genome duplication tends to favor the retention genes that are either highly expressed (Gout et al. 2010) or highly interactive, for example, through gene networks or protein–protein interactions (Lynch and Conery 2000; Papp et al. 2003). Although SITs do not appear to fit the latter category, as discussed in more detail below, expression constraints may have favored long-term retention of the SIT3 ohnolog, which is nevertheless dispensable based on our results.

Among the two principal SIT gene lineages (fig. 2), SIT1–2 genes are generally present in multiple copies in the genome and are known to actively transport DSi (Thamatrakoln and Hildebrand 2007), a function that has been preserved through purifying selection measured here and elsewhere (Alverson 2007; Thamatrakoln et al. 2006). In contrast, selection was relaxed in SIT3 genes, which have followed a much different evolutionary trajectory, that is, SIT3 has been lost repeatedly and is missing from nearly half of Thalassiosirales. Despite this, SIT3 is nevertheless present in species spanning nearly the full phylogenetic breadth of Thalassiosirales, so long-term conservation of SIT3 over the past 90–100 million years (Roberts et al. 2023) suggests that it might confer some benefit to the species that still have it. Different hypotheses about the function of SIT3 include a sensory role (Thamatrakoln and Hildebrand 2007) or a high-capacity, low-affinity transporter (Curnow et al. 2012).

Alternatively, the patterns observed here might be explained by a neutral model of ohnolog retention and loss. Under a model of absolute dosage subfunctionalization, a whole-genome duplication event doubles all the genes and their regulatory sequences (Gout and Lynch 2015), resulting initially in equal expression of ohnologs and, in this case, equal contributions to silicon transport. As long as the absolute levels of transcripts and protein products are maintained, the ohnologs can diverge neutrally in expression, with stochastic decreases in the expression of one ohnolog compensated for by increases in the other (Gout and Lynch 2015). As the imbalance increases over time, the ohnolog with the lowest expression level can be lost without negative fitness effects (Gout and Lynch 2015). For all but one of the species investigated here, the summed expression of SIT1–2 paralogs far exceeded the summed expression of SIT3 (fig. 4*a*). These differences, combined with the discoveries of relaxed selection on SIT3 sequences and recurrent losses over evolutionary timescales, together suggest that SIT3 continues to function in silicon transport, but its minimal and ever-dwindling contributions have made it expendable. Other diatoms have similarly divergent SIT types that might follow a similar model, though the mechanism of duplication and age of the duplicates are less clear. The diatom *Phaeodactylum tricornutum*, for example, has five SITs, one of which is expressed at low levels and was suggested to be either a pseudogene or to function in something other than silicon transport (Sapriel et al. 2009).

### Duplication, Loss, and Transcriptional Dynamics of SITs

Since their discovery (Hildebrand et al. 1997), it was clear that gene duplication has played an important role in the evolution and functional diversification of SITs (Thamatrakoln and Hildebrand 2007; Sapriel et al. 2009). The SIT1–2 lineage in particular has undergone an extensive history of duplication and loss, with many duplications mapping to terminal branches on the species tree (fig. 2). This pattern is similar to gene family dynamics in fungi, where genes encoding transporters and cell wall proteins had among the highest rates of duplication and loss (Wapinski et al. 2007). All available evidence suggests that the SIT1–2 type is responsible for most of the silicon uptake (Thamatrakoln and Hildebrand 2007), and most of the species in this study had multiple SIT1–2 paralogs, suggesting absolute dosage or dosage subfunctionalization plays key roles in the optimization of silicon uptake by diatoms. Most if not all SIT1–2 duplications were the products of small-scale duplication events, not genome duplications, which have been relatively rare in this group (Parks et al. 2018). Genes originating through small-scale duplications tend not to be part of large protein complexes or large networks, where duplication of a single gene disrupts the stoichiometric balance of interacting proteins (Lynch and Conery 2000; Maere et al. 2005; Papp et al. 2003). We hypothesize that small-scale SIT duplications are unlikely to have these types of deleterious consequences, so preservation–loss dynamics of new SIT duplicates should be governed principally by selection for or against increased dosage, which itself owes to any number of mostly unknown factors, such as silicon availability, cell size, or cell wall thickness in response to grazing pressure (Ryderheim et al. 2022)—the latter illustrating that all these factors have their own covariates.

SIT expression is best measured in a time series across the cell cycle and in synchronized cultures grown in low DSi, where SIT expression is highest and reflects changes in silicon demand by the diatom (Thamatrakoln and Hildebrand 2007). The measurements made here, in replete silicon, nevertheless showed strong within-species differences among transcript levels of SIT1–2 paralogs, suggestive of some degree of subfunctionalization. Follow-up experiments might show whether these differences are partitioned across the cell cycle, whether expression patterns are responsive to DSi availability, whether paralogs with low expression in our experimental conditions are highly expressed in others, or whether low expression might reflect early stages of silencing and loss. It is also possible that some SIT paralogs function primarily in DSi efflux, which occurs after the accumulation of excess soluble silicon in the cytoplasm to prevent autopolymerization inside the cell (Hildebrand 2008). The expression data presented here, together with the gene phylogeny, provide a framework to test these and other hypotheses about the functional consequences of duplication and loss of diatom SITs. Recent species-specific gene duplications also provide an opportunity to understand how quickly subtle changes in SIT expression can evolve in these species.

In addition to SITs, gene duplication and subfunctionalization appear to play a common and important role in the evolution of other transporter proteins in diatoms. The marine diatom *Cylindrotheca fusiformis* has two distinct ammonium transporters that differ in their expression, substrate affinity, and transport capacity (Hildebrand 2005). Also in *C. fusiformis*, two transporters for a different nitrogen source, nitrate, are expressed at similar levels, but like SITs, their expression levels covary predictably across the cell cycle (Hildebrand and Dahlin 2000).

### Relaxed Selection on Freshwater SITs

The rise of diatoms to their current prominence in modern oceans led to a global decrease in silicon availability in the marine environment (Conley et al. 2017). Average silicon concentrations in freshwaters (∼85 μM; Frings et al. 2014) are nearly an order of magnitude greater than the ocean (∼10 μM; Frings et al. 2016)—a disparity that has likely driven some of the well-known differences in silicon physiology between marine and freshwater diatoms. Marine diatoms have a greater affinity for DSi and more efficient uptake kinetics, and they require less silicon overall due to their comparatively thinner cell walls (Conley et al. 1989; Martin-Jézéquel et al. 2000). These traits are likely adaptive and make marine diatoms more competitive for trace amounts of environmental silicon in modern oceans. Diatom SITs cotransport sodium (Bhattacharyya and Volcani 1980; Knight et al. 2016) and/or potassium (Sullivan 1976) alongside DSi, so the greater availability of sodium in salt water might be more favorable to DSi transport as well.

We hypothesized that the relative surplus of DSi in freshwater systems released SITs from the strong selective constraints—measured here as strong purifying selection—to maintain the efficient, high-affinity SITs required to compete in marine environments. A previous study of SIT1–2 paralogs in Thalassiosirales rejected this hypothesis (Alverson 2007), but against a background of purifying selection, sites that have experienced bursts of episodic diversifying (positive) selection are not easily detectable by earlier site-based codon models (Murrell et al. 2012). This was illustrated here by the FEL method, which assumes constant selective pressure across sites (Kosakovsky Pond and Frost 2005) and found that > 90% of sites in the alignment were under purifying selection and none under positive selection. The MEME model (Murrell et al. 2012), however, showed that although purifying selection predominated, as many as 16% of sites had experienced episodic selection at one time or another. Episodically selected sites were present across the SIT protein structure, both major SIT clades and in marine and freshwater species, so there was no clear connection to SIT function, gene duplication, or the environment.

We further tested whether reduced competition for abundant DSi might have led to relaxed selection on freshwater SITs, with a focus on SIT1–2 paralogs involved in active DSi transport. Although rejected previously (Alverson 2007), the RELAX method allows for a more direct test of relaxed selection in focal (freshwater) branches compared with a set of reference (marine) branches (Wertheim et al. 2015). RELAX showed that purifying selection was stronger in marine than freshwater branches in the SIT1–2 clade. Although purifying selection still predominated in freshwater SITs, they were less constrained by selection than marine SITs. Although consistent with our hypothesis, it is unclear whether the degree of relaxation has important functional consequences and whether the relaxation was due to increased silicon availability, decreased sodium, or some other factor. Detailed functional studies, perhaps including genetic complementation of freshwater SITs into a marine species, would help resolve some of these questions. Other diatom lineages have traversed the marine–freshwater divide, and dense genomic sampling of one of these lineages, such as Surirellales (Ruck et al. 2016), would provide a strong but less direct test of the impact of freshwaters on SIT evolution. Finally, the SITs of marine and freshwater taxa were structurally similar, containing the same number of TMDs and the same conserved motifs, so key functional aspects of SITs were conserved across marine and freshwater taxa.

This study provides new insights into the evolution of an environmentally responsive and ecologically important gene family. Our results suggest that SIT sequence diversity, copy number, and gene expression have been shaped by small-scale and whole-genome duplications, neutral and adaptive processes, and environmental changes. The complex dynamics of SIT evolution, over time and across species, suggest that optimization of silicon uptake by diatoms is a difficult and probably moving target. In addition, the complex dynamics of SIT evolution make them a potentially valuable empirical system for testing general theories of gene expression and gene family evolution.

## Materials and Methods

### Data Set Construction and Phylogenetic Analysis

As described by Roberts et al. (2023), the predicted protein-coding genes from 37 draft genomes (25 marine and 12 freshwater) and assembled transcriptomes of four outgroups (*Bellerochea*, *Ditylum*, *Lithodesmium*, and *Eunotogramma*) were clustered with OrthoFinder (Emms and Kelly 2019), resulting in a single SIT orthogroup. Preliminary alignments and phylogenetic trees (see Materials and Methods below) were used to remove partial (<400 nt) or redundant sequences, based on zero- or near-zero branch lengths between SITs from the same genome. The final set of amino acid sequences was aligned with UPP, and the corresponding nucleotide coding sequences (CDS) were aligned by reconciling them against the amino acid alignment with translatorX (Abascal et al. 2010), resulting in an in-frame codon alignment. Columns masked by UPP and gap-rich columns identified by trimAl with the “-gappyout” setting were trimmed from the amino acid and codon alignments (Capella-Gutiérrez et al. 2009). The SIT gene phylogeny was inferred from the trimmed CDS alignment, which had a total of 117 sequences (108 ingroup and 9 outgroup) and 1,753 aligned columns, 1,451 of which were parsimony informative. The alignment was partitioned by codon position, and IQ-TREE (ver. 1.6.12) was used to identify the substitution model and partition scheme that provided the best fit to the alignment (‘-m TESTMERGE’) (Nguyen et al. 2015). The model included edge-proportional branch lengths (‘-spp’) to account for differences in evolutionary rates among codon positions. Branch support was based on 10,000 ultrafast bootstrap replicates (Hoang et al. 2018), and the tree search was run ten times independently to ensure recovery of the best possible tree topology. The final tree was rendered with the R-package ggtree (Yu et al. 2017). Alignments, program commands, and code are available in Zenodo repository 10.5281/zenodo.8036929.

### Gene Duplication and Loss

We used the gene tree/species tree reconciliation method implemented in the software package NOTUNG (ver. 2.9.1.5) to infer the history of gene duplication and loss in Thalassiosirales (Chen et al. 2000). The analysis takes two phylogenetic trees as input: a multilabeled SIT gene tree and a singly labeled Thalassiosirales species tree. In essence, NOTUNG embeds the SIT gene tree into the species tree to pinpoint gene duplications or losses (Chen et al. 2000). We removed the 8 most distant outgroup sequences from this analysis and retained the single nearest outgroup sequence from *E. lunatum*, resulting in a total of 109 leaf nodes (i.e., terminal branches) in the gene tree and 38 leaf nodes (i.e., species) in the species tree. NOTUNG reconciliation used default settings (edge weight threshold, 90.0; duplications, 1.5; losses, 1.0; codivergences, 0). The input files for this analysis are available in Zenodo repository 10.5281/zenodo.8036929.

### SIT Structure Prediction

We used the CCTOP prediction web server (Dobson et al. 2015) to predict the SIT protein topology (TMDs and internal and external loops) and positions of functionally important conserved motifs. Predictions were performed on the untrimmed amino acid sequences. CCTOP uses ten prediction methods and incorporates previously determined structural information from homologous sequences in the Topology Data Bank of Transmembrane Proteins database, which serves as a constraint on the query sequence (Dobson et al. 2015). We used TOPO2-Transmembrane (Johns and Speth 2010) to visualize the predicted secondary structure.

### Molecular Evolution

We fit a range of codon models, which measure the rates of nonsynonymous (dN, changes the amino acid) and synonymous (dS, does not change the amino acid) nucleotide substitutions to estimate the relative impacts of positive, negative, or relaxed selection on the evolution of diatom SITs (Kosakovsky Pond et al. 2005). These methods estimate the dN/dS ratio (ω) for each codon to identify the intensity of natural selection on both codons and branches in the SIT gene tree. Each codon is fit into one of three classes (ω < 1, ω = 1, ω > 1), which are indicative of purifying (negative) selection, neutral evolution, and diversifying (positive) selection, respectively (Yang 2002). Purifying selection (ω < 1) prevents the fixation of nonsynonymous mutations, constraining changes in protein function. If nonsynonymous mutations are relatively unconstrained by natural selection, the rates of dN and dS will be similar (ω = 1). Positive selection may promote the fixation of beneficial nonsynonymous substitutions, to optimize or diversify protein function, for example, resulting in higher rates of nonsynonymous substitutions (ω > 1) (Yang 2002). Because the latter scenario can represent a short-lived (episodic) change in the intensity of selection, codon models that average substitution rates across the phylogeny have lower power to detect these cases (Yang and Dos Reis 2011), motivating the development of models that test specifically for episodic selection. We fit a range of codon models implemented in HyPhy (Kosakovsky Pond et al. 2005) and run on the Datamonkey Adaptive Evolution Server (Weaver et al. 2018) to test different hypotheses about the role of selection in SIT evolution. We used HyPhy because its models incorporate site-to-site variation in the rate of synonymous substitution, which provides a better fit to most empirical data sets (Kosakovsky Pond and Muse 2005; Dimitrieva and Anisimova 2014) and reduces the type I error rate for these kinds of analyses (Wisotsky et al. 2020). In addition, HyPhy implements a range of models that allow us to specifically test the main hypotheses of our study.

### Tests of Positive Selection

Previous analyses showed that SITs evolve predominantly under strong purifying selection (Alverson 2007; Thamatrakoln et al. 2006). We used the FEL method, which estimates ω on a per-site basis and assumes constant selection pressure across the phylogeny (Kosakovsky Pond and Frost 2005). Among the site-based methods implemented in HyPhy, FEL is best suited for large phylogenies (Kosakovsky Pond and Frost 2005).

We used the BUSTED and MEME methods (Murrell et al. 2015, 2012) implemented in HyPhy to test for positive selection on SIT sequences. As a first test for positive selection, we used BUSTED, which is a gene-wide branch–site test that infers whether at least one site in a gene has experienced positive selection on at least one test branch, in our case the entire SIT phylogeny (Murrell et al. 2015). After finding evidence for episodic selection from BUSTED, we used MEME, a branch–site model that tests for episodic diversifying (positive) selection (Murrell et al. 2015). We used a chi-square test to test whether positively selected sites were distributed proportionally across internal loops, external loops, and transmembrane segments of the protein (fig. 1).

### Tests of Relaxed Selection

RELAX is a branch-based test to determine whether there was a detectable decrease in the strength of selection along a set of test branches compared with a set of reference branches (Wertheim et al. 2015). RELAX is a two-step test: first, a null model with three ω classes is fit to the gene tree, and in the second step, RELAX introduces a selection intensity parameter *k* (where *k* ≥ 0) as an exponent for ω values (ω^k^) estimated by the null model. RELAX fixes the inferred ω values and then fits an alternative model with ω^k^ on the test branches (Wertheim et al. 2015). Values of k > 1 indicate that selection was intensified on the test branches, whereas k < 1 indicates that selection was relaxed. RELAX is useful for identifying shifts in the stringency of natural selection on a gene tree but only relative to other parts of the tree.

Among many other variables, freshwater and marine environments have large differences in salt and silicon concentrations. Diatom SITs cotransport DSi and sodium (Bhattacharyya and Volcani 1980), suggesting that marine and freshwaters might present different opportunities and constraints on the evolution of diatom SITs. We used RELAX to test whether selection had been relaxed in freshwater SITs, due possibly to the comparatively high levels of DSi in freshwaters. We also tested whether selection had been relaxed in one SIT paralog (SIT3) that may play a reduced role in silicon transport.

### Transcription Profiles

We sequenced the transcriptomes of seven phylogenetically diverse species and measured SIT expression under optimal growth conditions. A total of eight replicates per strain were grown in a Percival incubator (15°C, 16:8 light:dark light regime, 22 μmol photons m^−2^ s^−1^ irradiance). All strains were grown in artificial seawater (Pinseel et al. 2022), with salinity adjusted to match the source environment of each strain. Experimental strains included *Conticribra weissflogii* AJA159-02 (0 ppt), *C. nana* CCMP1335 (32 ppt), *Skeletonema potamos* AJA081-03 (0 ppt), *C. invisitatus* AJA276-04 (0 ppt), *Stephanodiscus minutulus* AJA276-08 (0 ppt), *Discostella pseudostelligera* AJA232-27 (0 ppt), and *Thalassiosira pacifica* AJA261-08 (16 ppt). Cells were grown in 24-well plates and harvested during exponential growth. Cells were stored at −80°C until RNA extraction using Qiagen's RNeasy Plant Mini Kit, after which RNA quality and quantity were measured using a TapeStation 2200 (Agilent), a NanoDrop 2000c (ThermoScientific), and a Qubit 2.0 (Invitrogen). Library preparation and sequencing were performed by Arbor Biosciences using the myReads RNA-seq library prep kit and Illumina NovaSeq sequencing platform (2 × 150 paired-end reads).

To improve sequence quality, raw reads were corrected with Rcorrector (Song and Florea 2015) and trimmed using Trimmomatic ver. 0.36 (Bolger et al. 2014) with default options. The adapter-trimmed RNA-seq reads were mapped to the CDS files of the respective genomes for each strain with Kallisto ver. 0.43.1 (Bray et al. 2016). Using edgeR ver. 3.36.0 (Robinson et al. 2010) in R ver. 4.1.0, we filtered the data to only include genes with at least one count per million (CPM) in at least three samples, after which we used TMM normalization (i.e., weighted trimmed mean of the log expression ratios) to eliminate technical variation due to library size and composition (Robinson and Oshlack 2010). Finally, we extracted SIT genes from the TMM normalized data for visualization of gene expression variation between SIT paralogs.

## Supplementary Material

Supplementary data are available at *Genome Biology and Evolution* online (http://www.gbe.oxfordjournals.org/).

## Supplementary Material

evad212_Supplementary_Data

## Data Availability

Initial and final nucleotide and amino acid alignments, HyPhy input file (nucleotide alignment with the corresponding tree), NOTUNG input files (species and gene trees), and R codes are available at Zenodo repository 10.5281/zenodo.8036929. Genomes and transcriptomes have been deposited at National Center for Biotechnology Information (NCBI) under BioProject PRJNA825288.
