# Supplementary material for: Gene Duplication, Shifting Selection, and Dosage Balance of Silicon Transporter Proteins in Marine and Freshwater Diatoms
Source: Genome Biol Evol. 2023 Nov 23;15(12):evad212. doi: 10.1093/gbe/evad212 (PMC10700740; doi:10.1093/gbe/evad212)
Supplement: evad212_Supplementary_Data [file evad212_supplementary_data.docx]

Supplementary Tab. 1. Event summary of specie-specific gene duplications and losses inferred by NOTUNG.

| Species | Duplications | Losses |
| --- | --- | --- |
| Conticribra guillardii | 1 | 1 |
| Conticribra weissflogii | 2 | 0 |
| Cyclostephanos invisitatus | 2 | 2 |
| Cyclostephanos tholiformis | 0 | 1 |
| Cyclotella atomus | 2 | 1 |
| Cyclotella baltica | 4 | 1 |
| Cyclotella choctawhatcheeana | 1 | 1 |
| Cyclotella cryptica | 3 | 1 |
| Cyclotella distinguenda | 1 | 2 |
| Cyclotella kingstonii | 0 | 3 |
| Cyclotella meneghiniana | 1 | 1 |
| Cyclotella nana | 1 | 1 |
| Detonula confervacea | 0 | 2 |
| Discostella pseudostelligera | 0 | 1 |
| Discostella steligeroides | 1 | 0 |
| Discostella stelligera | 0 | 0 |
| Mediolabrus comicus | 0 | 1 |
| Porosira glacialis | 2 | 0 |
| Shinodiscus oestrupii | 0 | 1 |
| Skeletonema dohrnii | 1 | 1 |
| Skeletonema grethae | 0 | 1 |
| Skeletonema menzellii | 2 | 0 |
| Skeletonema potamos | 1 | 1 |
| Skeletonema tropicum | 0 | 0 |
| Stephanodiscus minutulus | 2 | 1 |
| Stephanodiscus triporus | 0 | 1 |
| Thalassiosira allenii | 0 | 2 |
| Thalassiosira delicatula | 1 | 0 |
| Thalassiosira exigua | 1 | 0 |
| Thalassiosira hispida | 0 | 1 |
| Thalassiosira livingstoniorum | 1 | 0 |
| Thalassiosira mediterranea | 2 | 0 |
| Thalassiosira oceanica | 2 | 2 |
| Thalassiosira ordinaria | 1 | 2 |
| Thalassiosira pacifica | 2 | 1 |
| Thalassiosira profunda | 2 | 1 |
| Thalassiosira sp. AJA248 18 | 1 | 2 |


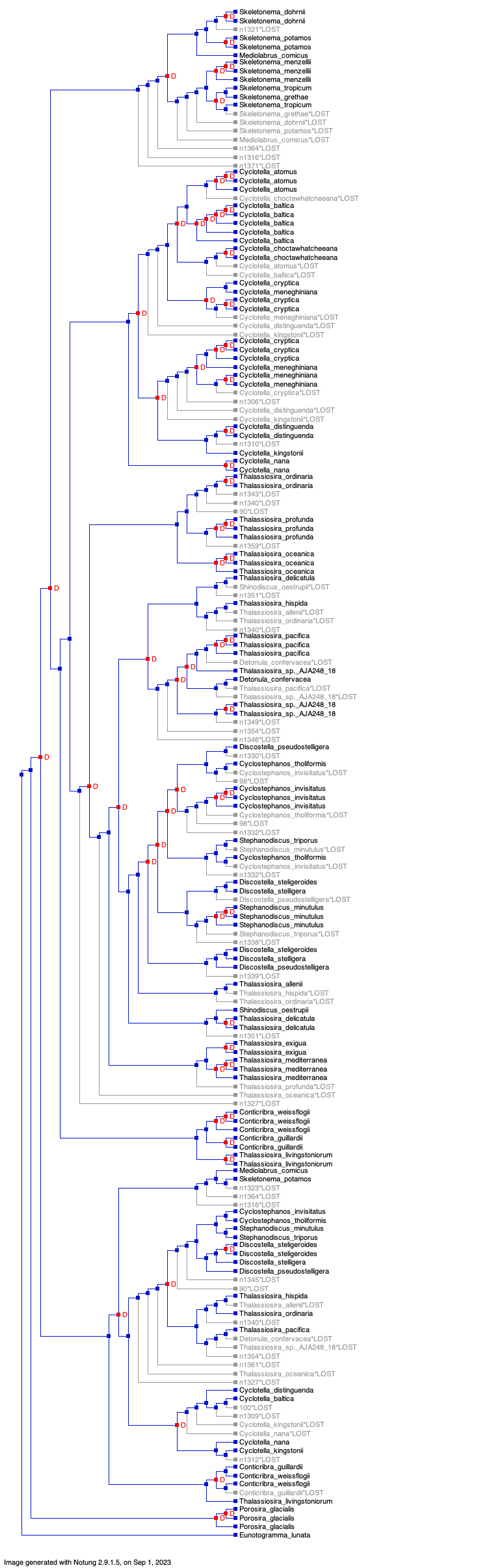


SIT 1-2

SIT 3

Supplementary fig. 1. Reconciled tree in NOTUNG format with marked internal and terminal gene duplications and losses.


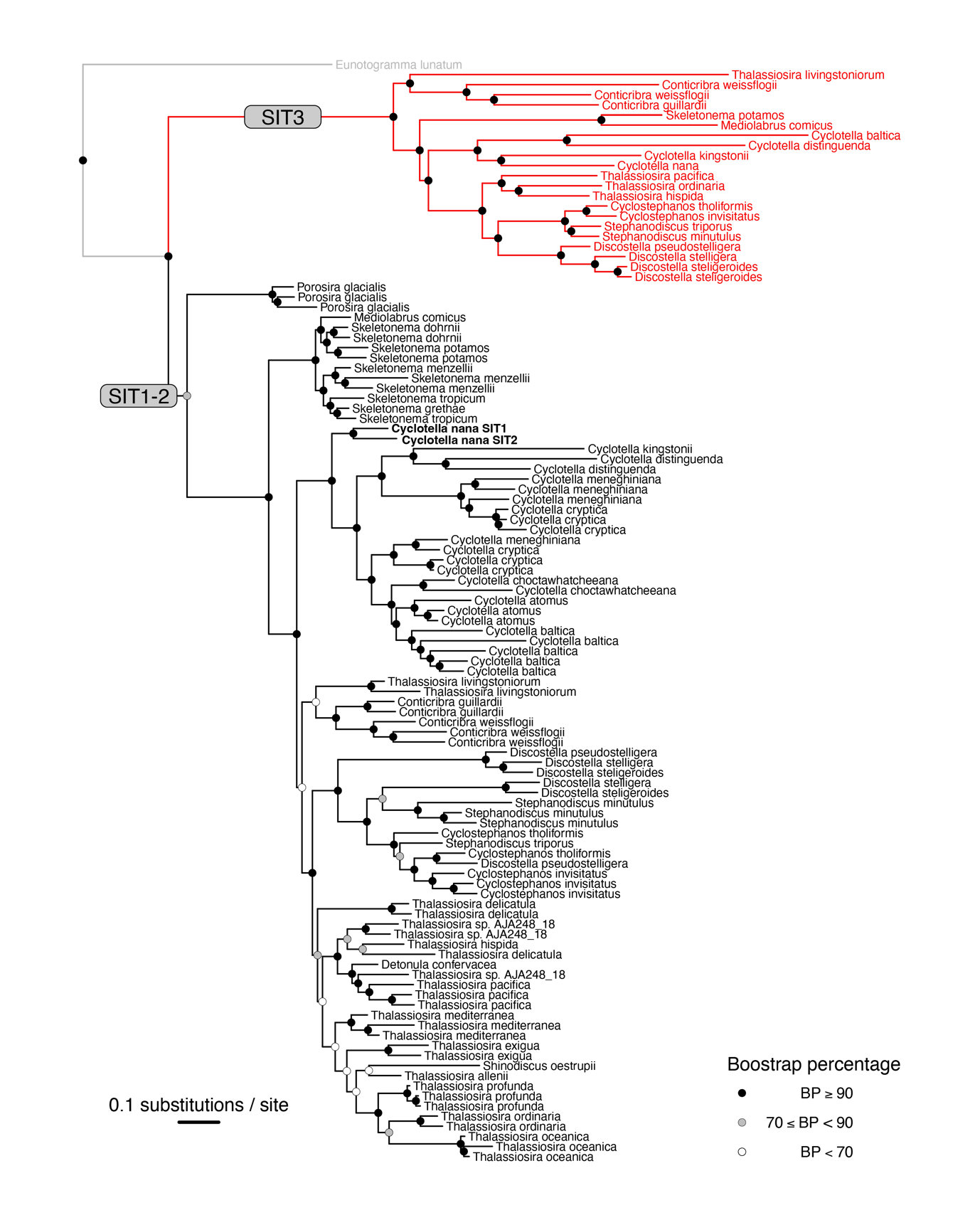


Supplementary fig. 2. RELAX test on the SIT3 clade. Foreground test branches are red, background reference branches are black, and branches excluded from the test are grey.


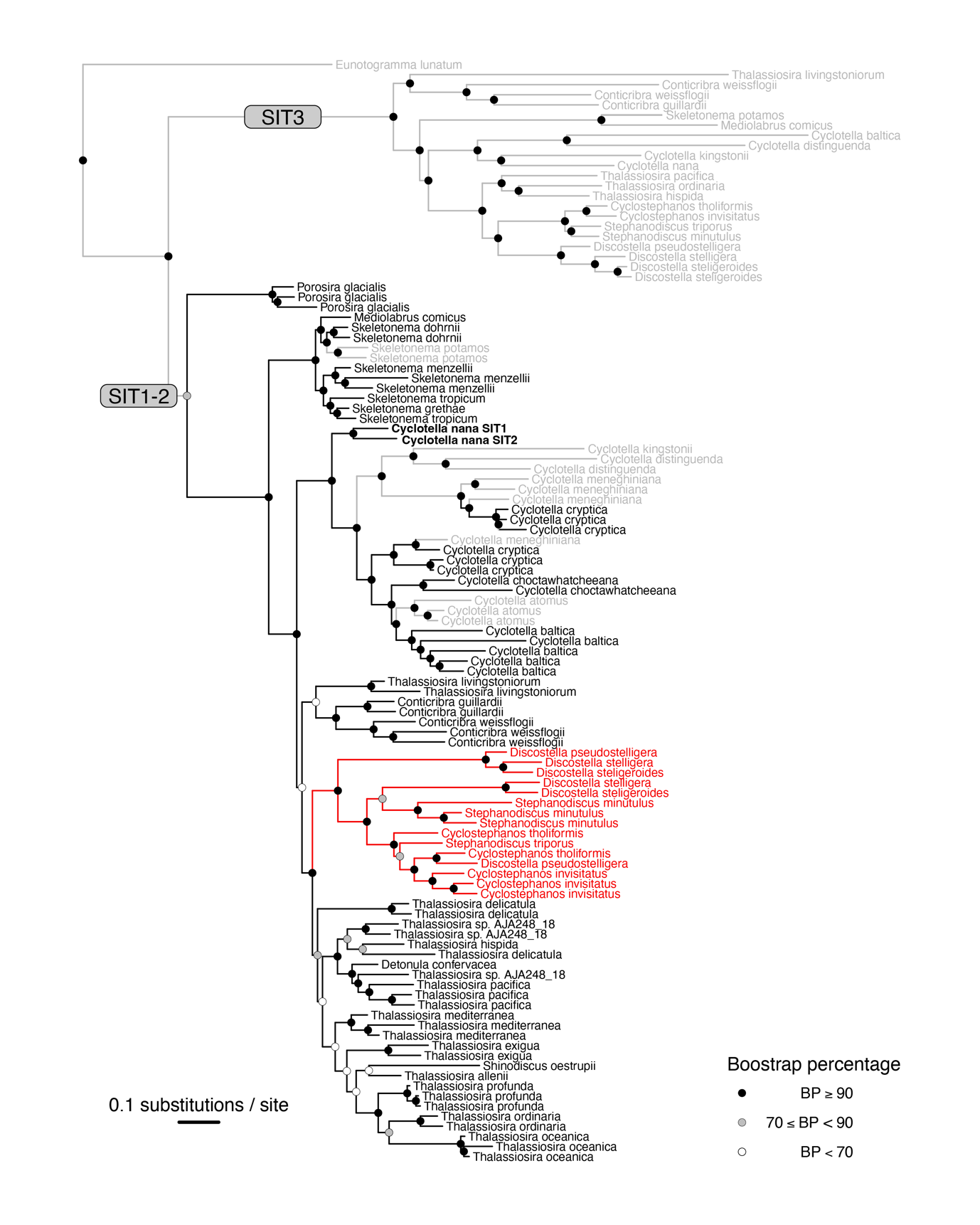


Supplementary fig. 3. RELAX test on SIT1-2 of cyclostephanoid clade. Foreground test branches are red, background reference branches are black, and branches excluded from the test are grey.

Supplementary fig. 4. RELAX test on freshwater SIT1-2 branches. Foreground test branches are red, background reference branches are black, and branches excluded from the test are grey.


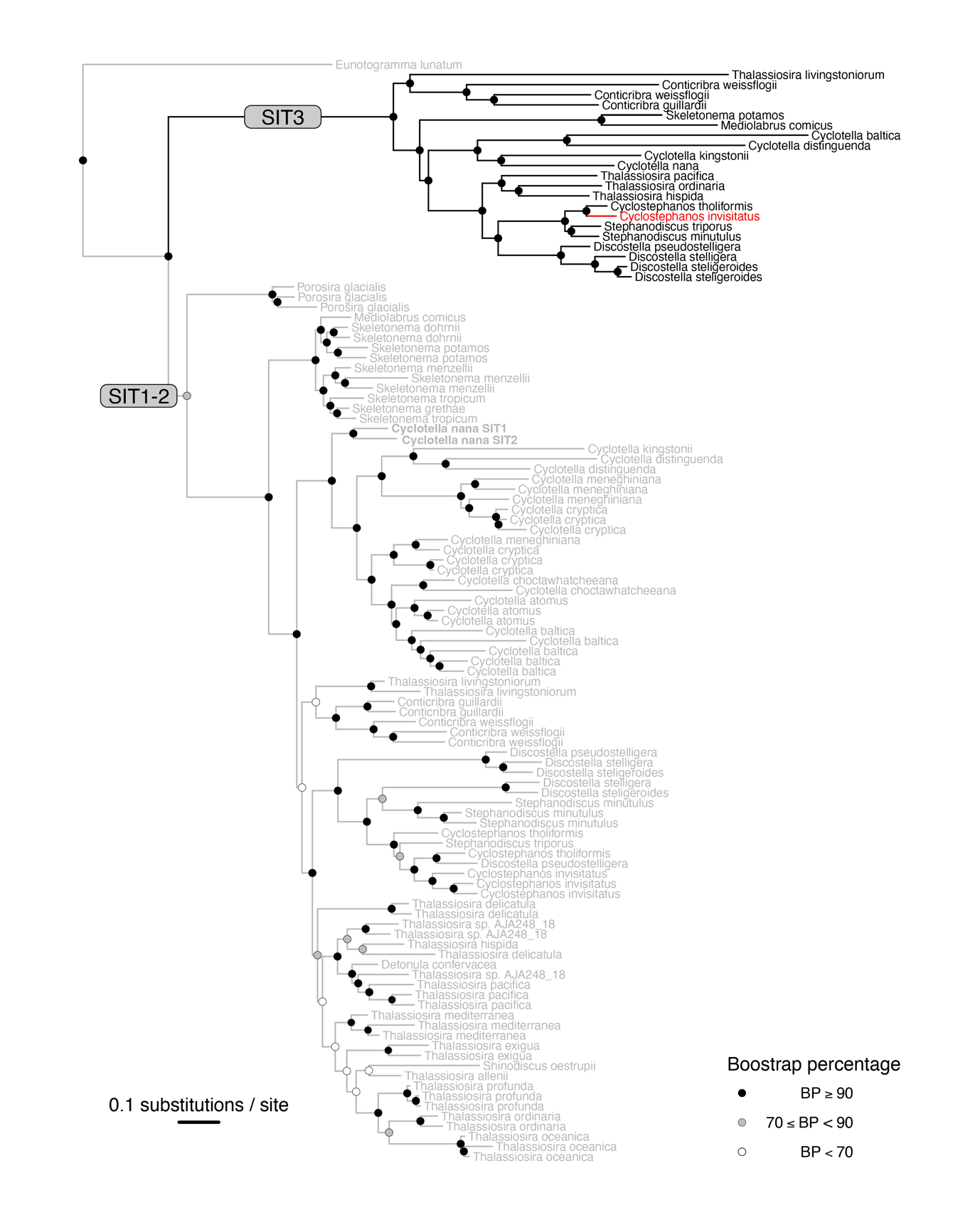


Supplementary fig. 5. RELAX test on SIT3 of *Cyclostephanos invisitatus*. Foreground test branches are red, background reference branches are black, and branches excluded from the test are grey.
